# Indentation Response of Calcium Aluminoborosilicate Glasses Subjected to Humid Aging and Hot Compression

**DOI:** 10.3390/ma14133450

**Published:** 2021-06-22

**Authors:** Xiangting Ren, Pengfei Liu, Sylwester J. Rzoska, Boleslaw Lucznik, Michal Bockowski, Morten M. Smedskjaer

**Affiliations:** 1 Department of Chemistry and Bioscience, Aalborg University, 9220 Aalborg, Denmark; xiangtingr@bio.aau.dk (X.R.); pli@bio.aau.dk (P.L.); 2 Institute of High-Pressure Physics, Polish Academy of Sciences, 01-142 Warsaw, Poland; sylwester.rzoska@gmail.com (S.J.R.); bolo@unipress.waw.pl (B.L.); bocian@unipress.waw.pl (M.B.)

**Keywords:** calcium aluminoborosilicate (CABS) glasses, indentation response, hot compression, humid aging

## Abstract

Aluminoborosilicate glasses find a wide range of applications, which require good mechanical reliability such as surface damage resistance. Calcium aluminoborosilicate (CABS) glasses have recently been found to exhibit so-called intermediate behavior in terms of their response to sharp contact loading. That is, these glasses deform with less shear than normal glass and less densification than anomalous glasses. This deformation mechanism is believed to give rise to high crack initiation resistance of certain CABS glasses. In order to further improve and understand the micromechanical properties of this glass family, we studied the indentation response of different CABS glasses subjected to two types of post-treatment, namely hot compression and humid aging. Upon hot compression, density, elastic moduli, and hardness increased. Specifically, elastic modulus increased by as much as 20% relative to the as-made sample, while the largest change in hardness was 1.8 GPa compared to the as-made sample after hot compression. The pressure-induced increase in these properties can be ascribed to the increase in network connectivity and bond density. On the other hand, the crack initiation resistance decreased, as the hot compression increased the residual stress driving the indentation cracking. Humid aging had only a minor impact on density, modulus, and hardness, but an observed decrease in crack initiation resistance. We discuss the correlations between hardness, density, crack resistance, and deformation mechanism and our study thus provides guidelines for tailoring the mechanical properties of oxide glasses.

## 1. Introduction

Oxide glass materials find various applications due to their high hardness, transparency, and chemical durability [1]. Their mechanical properties have in particular received widespread attention. This is because the low practical strength and fracture toughness limit the present and potential future applications. The low strength arises due to surface flaws, at which the stress concentrates and could lead to catastrophic damage [2], and the glasses also do not have a stable shearing mechanism. To enable the design of stronger and more damage-resistant oxide glasses, different intrinsic [3] and extrinsic post-treatment methods have been tried, including chemical strengthening to limit the formation and propagation of strength-limiting cracks through the creation of a surface layer with high compressive stress [4].

Indentation testing can, to some extent and for certain applications, be used to simulate real-life damage events under controlled conditions. This is because sharp contact is often the main failure mode of glasses. The sample area required for indentation testing is relatively small and the experiment time is short. In terms of their Vickers indentation behavior, oxide glasses can be classified into “normal”, “anomalous”, or “intermediate”. The normal glasses deform by a shearing mechanism and form median/radical and lateral cracks, while anomalous glasses mainly deform through densification and often form ring/cone cracks. The intermediate glasses have some characteristics of typical normal and anomalous glasses, as they deform with less shear than normal glass and less densification than anomalous glass, but do not have ring/cone and median cracks [5]. The indentation deformation mechanism (e.g., degree of shear flow and densification) therefore depends to a large extent on the glass composition. Since densification is an effective way to dissipate the elastic energy applied to the material in the process of indentation, there has been an increasing interest in tailoring the deformation mechanism and thus cracking behavior through rational composition design.

The number of possible oxide glass compositions is extremely high [6] and outside the range of what could be tested experimentally, at least with the current methods for composition–structure–property studies. In the effort to improve the indentation response of oxide glasses, it is thus important to establish relationships between glass composition, indentation deformation mechanism, and the resulting indentation response. Knowledge of such relationships will advance the design of tailored oxide glasses, with the goal to shift the field from trial-and-error to model-based glass design. To enable this, it is important to understand at the atomic-scale the structural rearrangements that occur due to the high stress levels induced by indentation. For instance, Lee et al. [7] showed that for vitreous boric oxide at room temperature, the average coordination number of boron increases with pressure, with irreversible changes starting at a pressure of 4–7 GPa. This pressure level is usually achieved in indentation experiments.

In this study, we focused on the indentation response of a family of calcium aluminoborosilicate (CABS) glasses. They are interesting, as they have been found in recent work to exhibit intermediate indentation behavior, i.e., they deform with higher densification than typical normal glasses and also more shear deformation than anomalous glasses [8]. This gives rise to a high crack initiation resistance during indentation, which in turn has been related to high shear band density in some CABS glasses. In general, such aluminoborosilicate glasses can be applied as flat panel display substrates, photochromic components, bioactive materials, and nuclear waste encapsulation materials [9]. The network connectivity of the intermediate CABS glasses is lower than that of anomalous glasses, which leads to the greater degree of shear deformation, allowing them to be relieved from the stresses that form ring and median cracks [10]. However, in the studied CABS glass series with varying SiO_2_/B_2_O_3_ ratios [6], the relative amounts of shear and densification deformation were constant, but the load leading to indentation cracking varied depending on the chemical composition. Therefore, in this work, we aimed to further explore and understand the structure and composition dependence of the indentation response of CABS glasses. In CABS glasses, SiO_2_ and B_2_O_3_ are the basic network formers, due to the low cation size and high bond strength. Boron is found in either a three- or four-fold coordinated state with oxygen in a random configuration [11]. Both four-fold coordinated boron and aluminum need to be stabilized by calcium cations, or alternatively through the formation of five- or six-fold coordinated aluminum or oxygen triclusters. The addition of alumina to borate glass thus changes the boron speciation [12].

Besides composition optimization, we explored the effects of post-processing methods on the mechanical properties of CABS glasses. These methods included hot compression and humid aging treatment. First, heating the glass at a temperature close to the glass transition temperature (*T*_g_) under elevated pressure (i.e., so-called hot compression) can be used to permanently change the structure and properties of oxide glasses [13]. That is, hot compression at pressures of 1–2 GPa around *T*_g_ is known to increase density, hardness, and elastic moduli, whereas crack initiation resistance decreases [14,15,16]. In addition to being an important method that could help to tune the properties, compression experiments can provide insights into the indentation deformation mechanism due to the high stress that can be generated in glasses under sharp contact loading [17,18,19]. Second, humid aging treatment has been found to influence the mechanical properties. For example, Kim et al. studied the subsurface damage of soda–lime–silica float glass before and after hydrothermal treatment [20,21]. The glass–water reactions were accelerated at the subsurface damage layer. Interestingly, compared with the original float glass, the surface of hydrothermally treated glass exhibited lower hardness, higher crack resistance, and lower resistance to mechanochemical wear under higher humidity. Another study explored the influence of relative humidity during mechanical testing on the crack resistance of an alkaline earth aluminosilicate glass. Vickers hardness was not significantly influenced by the environmental conditions, whereas crack resistance decreased with the increase of humidity [22]. Recently, it was found that a cesium aluminoborate glass exhibits excellent crack resistance after being subjected to humid surface aging prior to indentation testing [23]. Such water entry into the glass surface may exert compressive stresses in the contact area [24,25], which could help to suppress the formation and propagation of cracks. In addition, the diffusion of water into the glass network may lead to rapid stress release [26]. However, the aging of a lithium aluminoborate glass [27] does not cause the ultra-high crack resistance values as for the cesium aluminoborate glasses. There is thus a need to understand the composition dependence of the effect of humid aging on glass mechanics. Considering their high inherent crack initiation resistance, performing humid aging treatment on CABS glasses is particularly interesting.

In this work, we used the 15CaO-15Al_2_O_3_-25B_2_O_3_-45SiO_2_ (named CABS-ref) glass from the previous study [6] as the reference composition, since it showed the highest crack initiation resistance and it was thus interesting to investigate if it could be further improved. We then performed five systematic composition variations in 5 mol% oxide increments, as shown in Table 1: CABS-SiB (Si/B increase), CABS-CaB (Ca/B increase), CABS-BAl (B/Al increase), CABS-CaSi (Ca/Si increase), and CABS-CaAl (Ca/Al increase). We performed hot compression and/or humid aging treatment on the six glasses and characterized their mechanical properties through ultrasound echography and micro-indentation. The indentation deformation mechanism was also explored.

## 2. Materials and Methods

### 2.1. Sample Preparation

We prepared the six calcium aluminoborosilicate (CABS) glasses using traditional melt quenching technology, with the nominal chemical compositions given in Table 1. The raw materials were CaCO_3_ (99.5%, ChemSolute, Renningen, Germany), Al_2_O_3_ (99.5%, Sigma-Aldrich, Seelze, Germany), H_3_BO_3_ (≥99.5%, Honeywell International, Schnelldorf, Germany), and SiO_2_ (≥99.5%, 0.2–0.8 mm, Merck KGaA, Schnelldorf, Germany). First, these are weighed and thoroughly mixed based on the target composition. To remove H_2_O and CO_2_, the mixed batch was gradually added to a crucible (Pt–Rh) in an electric furnace at 800 °C. Depending on the composition, these mixtures were then melted at 1600 °C. The melt was then poured onto a steel plate for quenching and then transferred to the annealing furnace at the glass transition temperature (*T*_g_). The actual *T*_g_ values were later determined from differential scanning calorimetry measurements (STA 449 F3 Jupiter, Netzsch, Selb, Germany) at 10 K/min. *T*_g_ values are summarized in Table 1 and also in Table S1 along with other property data. After these measurements, the glasses were reannealed at their measured *T*_g_ values for another 0.5 h and then cooled to room temperature with the cooling rate of about 3 K/min. After re-annealing, we cut the glasses into the required size for the subsequent characterization. Diamond grinding disks were then used to polish the samples in ethanol. Based on X-ray diffraction analyses (Empyrean XRD, PANalytical, Stanford, CA, USA) of all samples, no signs of crystallization were present (Figure S1 in the Supplementary Materials).

For the hot compression treatment, the glasses were isostatically compressed in a 1.0 GPa N_2_ atmosphere at their respective *T*_g_ values. The high temperature and pressure were maintained for 30 min, and subsequently the samples were cooled to room temperature at a cooling rate of 60 K/min to achieve permanent compression of the glass samples. The pressure chamber was then decompressed at a rate of 30 MPa/min. The humid aging treatment of all polished glass samples was done using an autoclave. Both as-made and compressed samples were subjected to this treatment. The conditions of the autoclave were at 120 °C and relative humidity of 100%. The treatment was carried out for a duration of 24 h, whereafter the temperature of the autoclave has dropped to room temperature and the glass sample was removed for relevant experimental tests.

### 2.2. Characterization

The density (*ρ*) values of the as-prepared and post-treated glasses were determined by Archimedes’ principle of buoyancy. The weight of each sample (at least 1.5 g) was measured 10 times in ethanol.

An ultrasonic thickness gauge (38DL Plus, Olympus, MA, USA), equipped with 20 MHz delay line transducers for the determination of the longitudinal *V*_1_ and transversal wave velocities *V*_2_, was used to determine the elastic properties of the glasses. Based on these velocity and density values, we calculated Young’s modulus *E*, bulk modulus *B*, and shear modulus *G* as well as Poisson ratio *ν* using the relations for isotropic materials.

We also calculated the atomic packing density (*C_g_*). To do so, we assumed four-fold coordination for Si, six-fold coordination for Ca, two-fold coordination for O, while the speciation for boron and aluminum was based on previous structural data for the CABS glasses [6]. *C_g_* is defined as the ratio between the theoretical molar volume provided by the ions and the effective molar volume of the glass. The atomic packing density (*C_g_*) can then be calculated as,

(1) $$C_{g}=\rho \frac{\sum f_{i}V_{i\;}}{\sum f_{i}M_{i}}\;,$$

where $V_{i}=\frac{4}{3}\pi N\left(xr_{A}{}^{3}+yr_{B}{}^{3}\right)$ represents the molar volume of an oxide A_x_B_y_ with the molar mass *M_i_* and the molar fraction *f_i_*, *N* is the Avogadro number, and *r_A_* and *r_B_* are the ionic radii [28,29,30].

Micro-indentation measurements were carried out with a Nanovea CB500 hardness tester to determine the Vickers hardness (*H*_V_) and crack resistance (CR). On each sample, 20 indentations with a maximum load of 4.9 N (1 kgf) were generated to determine *H*_V_, with a loading duration of 15 s and a dwell time of 10 s. We then used an optical microscope to analyze the residual imprints and calculate the *H*_V_. CR, i.e., the resistance of the glass to the initiation of cracks in the corners upon indentation, was determined using two different diamond indenters, namely, the 136° four-sided pyramid Vickers tip and the three-sided pyramid cube corner tip with mutually perpendicular faces. For the same glass, the sharper cube’s corner tip leads to higher residual stress, less densification, and easier crack initiation compared to the Vickers tip. In both cases, we used increasing loads (from 4.8 to 30 N for Vickers and from 0.1 to 0.9 N for cube corner) and counted the numbers of corner cracks 2 h after unloading (see Figure S2). CR was calculated based on the method of Wada [31]. That is, the probability of crack occurrence (PCI) was defined as the ratio between the number of corner cracks and the total number of corners on all indents. CR is a load that generates 2 or 1.5 cracks (PCI = 50%) on average for Vickers and cube corner, respectively. At least 30 indents were performed on each sample using a loading duration and dwell time of 15 s and 10 s, respectively. Measurements were performed under laboratory conditions (room temperature, relative humidity ~37% RH).

To better understand the indentation deformation mechanism (i.e., the relative propensity for densification vs. shear flow) in the different glasses, we explored the recovery of the indent side length. This analysis consists of recording images of the indent site before treatment and after a thermal treatment at 0.9 *T*_g_ for 2 h [32], and then measuring how much the side length of the indent cavity shrinks after the annealing treatment. Such side length recovery is in turn related to the degree of densification upon indentation, as the densified region will recover during the 0.9 *T*_g_ annealing but not the displacement due to shear flow. We explored at least 10 indents with the load of 4.9 N for each specimen, loading duration of 15 s, and dwell time of 10 s. The side length recovery (*L_SR_*) can then be calculated as,

(2) $$L_{SR=}\frac{L_{s,i-L_{s,f}}}{L_{s.i}}\;,$$

where *L_s,i_* is the indentation side length as defined from the optical microscope before the treatment and *L_s,f_* is the indentation side length after annealing at 0.9 *T*_g_ for 2 h.

## 3. Result and Discussion

### 3.1. Density and Elasticity

Table 2 summarizes the density results for the CABS glasses before and after the different post-treatments. First, considering the as-prepared (annealed) glasses, we found that the density was generally higher for the glasses with 20 mol% CaO (CABS-CaB, CABS-CaSi, CABS-CaAl) than those with 15 mol% CaO (CABS-ref, CABS-SiB, CABS-BAl). This is likely because the modifying Ca^2+^ ions occupy open spaces in the network, as also seen from the variation in the atomic packing density showed in Table S2 in the Supplementary Materials. When the content of CaO was constant, the change in the proportion of Al_2_O_3_ and B_2_O_3_ basically had no effect on density of the glass system. Upon hot compression, we found that the density increased, consistent with the results of other oxide glass systems [15,33,34,35,36]. We quantified the extent of this increase by calculating the so-called plastic compressibility (*β*_hot_) as −(1/*V*)(d*V*/d*p*) where *V* is volume and *p* is applied pressure (see Table 2). The reference CABS glass had the highest value of *β*_hot_. In particular, the addition of CaO and the removal of B_2_O_3_ and Al_2_O_3_ had a negative effect on *β*_hot_, likely because there is less room for volume densification in these more-packed glasses with fewer B and Al that can undergo coordination number changes [2]. Generally, we found that the humid aging treatment only had a minor impact on density for both as-made and compressed samples.

Table 3 indicates the composition and the post-treatment dependence of Young’s modulus (*E*), which is the resistance of the glass to elastic deformation along the axis when the opposite force is applied along the axis. *E* increased following hot compression for all these CABS glasses, consistent with previous findings [16]. *E* increased up to 14 GPa, which was an increase of ~20% relative to the as-made sample. This was ascribed to the increase in atomic packing density and the increase in connectivity of the glass network. That is, in the compressed glass more bonds are stretched or broken per unit volume upon loading, which increases the resistance to deformation and results in an increase in *E* [12]. It can be seen from Table 3 that the changes for samples with different components in Young’s modulus were similar to those for density (Table 2), and the changes in components also had an effect on the elastic modulus. Table 3 also demonstrates that the changes in *E* for the CABS glasses with the process of surface aging were negligible (i.e., the value was similar to those of the as-made glass).

### 3.2. Glass Transition Temperature

The glass transition temperature values were determined from differential scanning calorimetry measurements (Figure S3). Among the different glasses, CABS-CaB had the highest *T*_g_ value (683 °C) and CABS-BAl had the lowest *T*_g_ value (636 °C) (Table 1). Considering the CABS-CaB glass, the ratio of CaO and B_2_O_3_ increased relative to CABS-ref. Upon addition of more alkaline earth oxide into glass, more of the boron atoms will be converted from a three- to a four-coordinated [37,38,39]. As discussed above, the addition of four-coordinated boron results in a more rigid glass network, which can explain the higher *T*_g_ [40,41]. For the CABS-BAl glass, the content of Al_2_O_3_ is lower and that of B_2_O_3_ is higher, and the corresponding *T*_g_ value decreased accordingly. Among all the samples, the B_2_O_3_ content in CABS-BAl was the largest (30 mol%). The high total content of B_2_O_3_ in this glass and therefore also high amount of three-fold coordinated B makes the glass less rigid. ^[3]^B has a planar and open structure as it does not require charge compensation. This can explain the lower *T*_g_ [40,41].

### 3.3. Hardness

**Figure 1:**
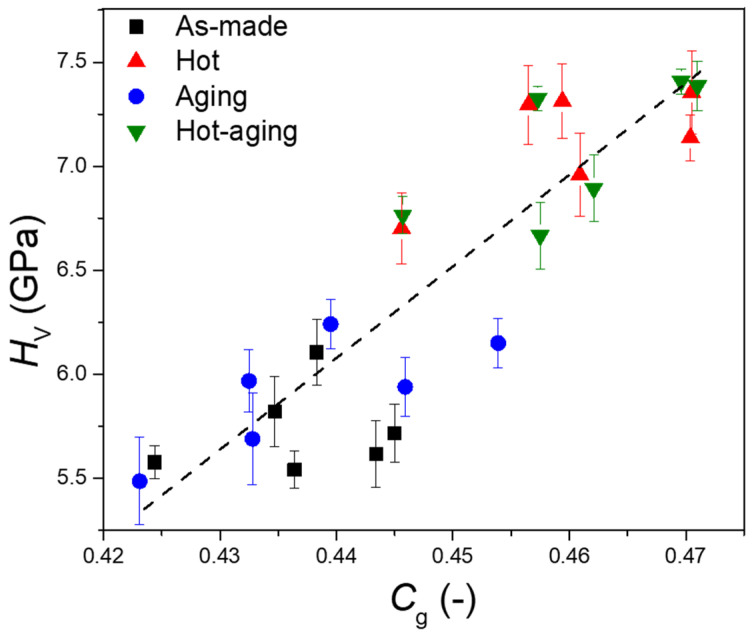
Relationship between Vickers hardness (*H*_V_) and atomic packing density (*C_g_*) of the CABS glasses subjected to different post-treatment. The dashed line is a guide to the eye.

As shown in Table 4, there were only minor differences in the Vickers hardness (*H*_V_) of the as-made CABS glasses with different composition. However, it was found that the Vickers hardness (*H*_V_) of all samples increased after hot compression. That is, *H*_V_ increased from 5.8 to 7.0 GPa, 5.5 to 7.3 GPa, 5.6 to 7.4 GPa, 5.6 to 6.7 GPa, 5.8 to 7.1 GPa, and 6.1 to 7.3 GPa for CABS-ref, CABS-SiB, CABS-CaB, CABS-BAl, CABS-CaSi, and CABS-CaAl samples, respectively. Hardness increased by up to 1.8 GPa upon hot compression compared to the as-made sample. We also note that *H*_V_ exhibited the same pressure dependence as density, consistent with previous research [42] and indicating that the densification of the overall network is also the reason for the increase in hardness during compression. In addition, it is known that the coordination number of boron and aluminum increases after hot compression, which results in more network bonds per atom. The increase in bond density and network connectivity is the reason for the increase in hardness induced by pressure [43]. Indeed, we found an approximate positive correlation between the atomic packing density and hardness (Figure 1). For the samples subjected to humid surface aging treatment, hardness remained almost unchanged compared with the as-made samples. This finding was also supported from the comparison between the compressed samples and the samples with combined treatment of hot compression and surface aging. The possible reason may be that there is little change or influence of surface aging on the internal structure of the glass. Since the hardness testing was performed at a load of 4.9 N, the indenter penetration depth was around 5 µm for these samples.

### 3.4. Crack Resistance

Indentation-induced crack initiation occurs under a sufficiently high load due to the mismatch between the amount of plastic deformation and the surrounding elastically deformed material. We used the indentation data generated at different loads to calculate the crack resistance (CR), which is defined as the load that causes two corner cracks to form per indent for the four-sided pyramid indenter. We note that corner cracks were the main type of cracks in the investigated glass. Different experimental conditions (such as loading rate and relative humidity) and composition changes will have a significant impact on the CR value. Therefore, compared with the starting glass CABS-ref, samples with different compositions also had different values of CR.

**Figure 2:**
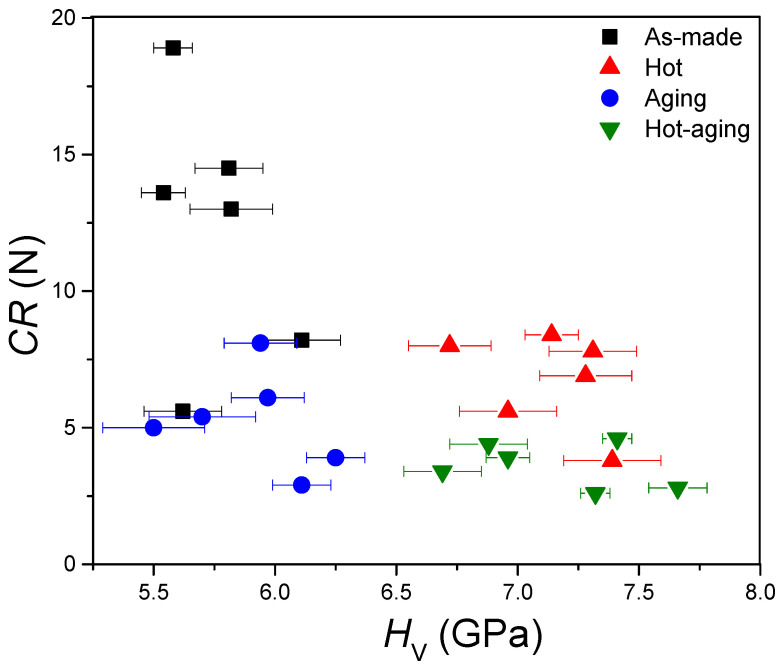
Relationship between crack resistance for Vickers indentation (CR) and hardness (*H*_V_) of the CABS glasses subjected to different post-treatment.

Among the CABS glasses, CABS-BAl had the largest CR value (18.9 N) and CABS-CaB had the lowest CR value (5.6 N) for Vickers indentation (Table 5). As discussed previously, the CABS-CaB glass has a more rigid structure that gives rise to more residual stress and thus a lower CR value. This is also seen from Figure 2 that shows the relation between CR from Vickers indentation and Vickers hardness. As the hardness increased, the crack resistance generally showed a decreasing trend. The results generally show that changing the fraction of CaO had a pronounced input on the mechanical properties, which in turn depended on the balance between the atomic bonding energies, the packing efficiency of atoms, and the ability of the network to structurally rearrange or densify. Therefore, after adding modified oxides (such as CaO), in addition to converting the three-fold coordinated B into four-fold coordinated B, the interstices are filled with modified cations. This is expected to partially hinder the indentation-induced densification and cause the glass to be more prone to deform through shear flow [44]. Alkaline earth modifier cations such as Ca^2+^ form relatively strong ionic bonds with oxygen, which also leads to higher hardness and modulus compared to alkali cations [45,46]. For the CABS-BAl glass, the ratio of B_2_O_3_ to Al_2_O_3_ increases and the glass thus have more three coordinated B atoms as discussed previously. As crack resistance is known to increase with increasing ^[3]^B content [47], it explains the high CR value of this glass. The positive effect of ^[3]^B_2_O_3_ on CR is due to the relatively high single bond strength [48], with ^[3]^BO (499 kJ/mol) > AlO (293–423 kJ/mol) [49]. Moreover, ^[3]^B has an open structure as it does not require charge compensation, and ^[3]^B also has a planar structure, which makes it easier to densify and increase its coordination number during indentation.

For the glasses subjected to the sharper cube corner tip (Table 6), the startingglass (CABS-ref) had the largest value of CR and CABS-BAl surprisingly had the smallest CR. This must be ascribed to different deformation mechanisms as the tip sharpness changes [5]. That is, when the tip is sharper, the glass is not able to densify as much as with the blunter Vickers tip. Therefore, the main advantage of the CABS-BAl glass (i.e., its structural densification ability) disappears.

Upon subjection of the glasses to compression at *T*_g_ and 1 GPa, the residual stress that drives the indentation cracking is higher than that of the as-made glass. This leads to more pronounced cracking. Therefore, the material cannot dissipate mechanical energy through densification, resulting in lower CR values under both indenter tips (Table 5 and Table 6). After the humid aging treatment of the samples, the CR values of CABS glasses all decreased. On the one hand, in less chemically durable aluminoborate glasses, it has been found that CR can increase upon humid aging, for example due to a compressive stress layer [5]. On the other hand, it has been found that water can facilitate crack formation in some silicate glass [34,35]. Earlier research has also suggested that water may be causing a weakening of the glass network [50,51,52]. Water could also initiate cracks from a blunt crack under a subcritical stress while other liquids cannot. In the relatively durable CABS glasses, it is therefore expected that CR decreases after humid aging in the relatively low-RH atmosphere. Consequently, the combined treatment of hot compression and humid aging also decreases the CR values of these CABS glasses.

### 3.5. The Recovery of the Indentation Side Length (L_SR_)

**Figure 3:**
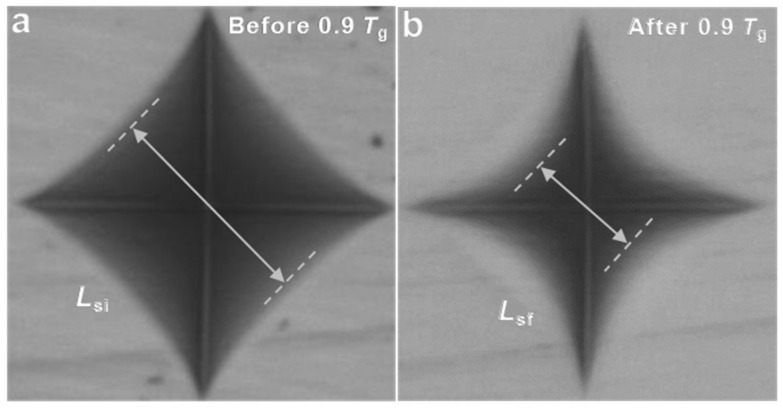
Optical micrographs of indents generated at 4.9 N on the surface of the CABS glass before and after re-annealing at 0.9*T*_g_ for 2 h.

Table 7 shows the composition dependence of indentation side recovery (*L*_SR_) for the present as-made glasses. *L*_SR_ is a measure of the degree of shrinkage of a Vickers indent as caused by a thermal treatment below *T*_g_, and previous studies have shown that *L*_SR_ can be used as a measure to determine the densification contribution to indentation deformation [47]. As seen in Figure 3, the indent had partially recovered its shape after annealing. CABS-BAl had the largest *L*_SR_ value (37.3%), while CABS-CaB had the lowest *L*_SR_ value (29.7%) relative to the *L*_SR_ value of CABS-ref (35.9%). Considering the CABS-BAl glass, it may be explained by the higher proportion of B_2_O_3_ and thus concentration of three-fold coordinated B atoms. The open planar structure of these made it easier for the glass to densify during the indentation process and thus resulted in a higher *L*_SR_. The low *L*_SR_ value of CABS-CaB may be because the addition of CaO converted more of the three-fold coordinated boron atoms into four-fold coordinated boron. This resulted in a more rigid structure, with less densification during the indentation process.

**Figure 4:**
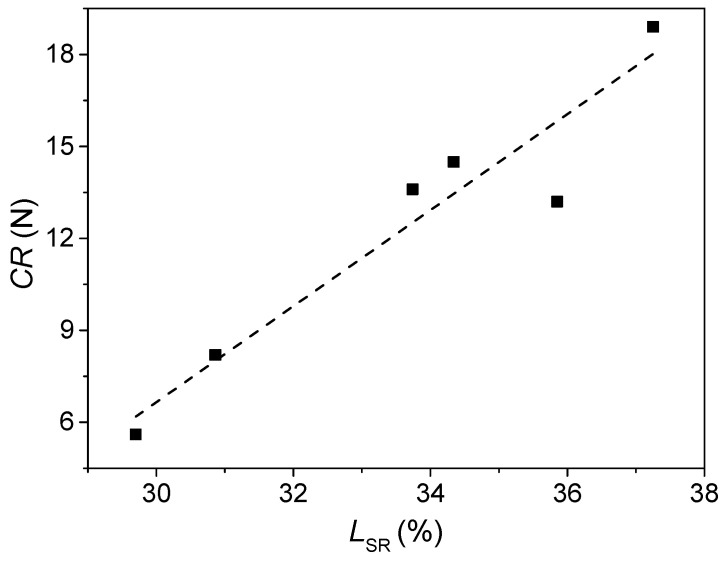
Relationship between crack resistance for Vickers indentation (CR) and indentation side recovery (*L*_SR_) of the as-made CABS glasses.

In general, CABS glasses with lower CaO content (15%) had a larger identification side recovery (*L*_SR_) value. This is also similar to the variation in CR. Figure 4 shows how CR was generally positively related to the degree of recovery and thus the extent of densification. This is in agreement with previous studies, showing that densification is favorable for increasing the crack initiation resistance as it allows the glass network to lower the residual stress, i.e., the driving force for the crack initiation.

## 4. Conclusions

We studied the mechanical properties of a series of calcium aluminoborate glasses (CABS) with different composition ratios and subjected these to two types of post-treatment, namely hot compression and humid aging. For the glasses subjected to an isostatic compression treatment at their respective *T*_g_ at the pressure of 1.0 GPa, the density, elastic moduli, and hardness increased. Young’s modulus increases by up to 20% relative to the as-made sample, which was mainly due to the increase in atomic packing density. Hardness increased up to 1.8 GPa compared to the as-made sample after hot compression, which was ascribed to the increase in network connectivity and bond density. On the other hand, the crack initiation resistance decreased, as the residual stress driving the indentation cracking was higher compared in the hot compressed glass than that in the as-made glass. Upon the treatment in a humid atmosphere, only minor effects on density, modulus, and hardness were observed, whereas the crack resistance decreased. Therefore, we can adjust the mechanical properties by changing the composition, as well as through the treatment of hot compression and humid aging. We observed positive relations between hardness and atomic packing density on one hand and crack resistance and the extent of densification on the other, while hardness and crack resistance appeared to be negatively correlated. These approaches thus provide a means to tailor the mechanical properties of glasses.

## Figures and Tables

**Table 1:** Nominal chemical compositions of the CABS glasses as well as their measured glass transition temperature (*T*_g_). Coloring indicates reference content or *T*_g_ (grey), increasing content or *T*_g_ relative to reference (green), and decreasing content or *T*_g_ relative to reference (red). The error in *T*_g_ was within ±2 °C.

Glass ID	SiO_2_ (mol%)	Al_2_O_3_ (mol%)	B_2_O_3_ (mol%)	CaO (mol%)	Note	*T*_g_ (°C)
CABS-ref	45	15	25	15	Reference glass	677
CABS-SiB	50	15	20	15	Si/B increase	652
CABS-CaB	45	15	20	20	Ca/B increase	683
CABS-BAl	45	10	30	15	B/Al increase	636
CABS-CaSi	40	15	25	20	Ca/Si increase	668
CABS-CaAl	45	10	25	20	Ca/Al increase	665

**Table 2:** Density (*ρ*) of the as-made, hot-compressed, humid-aged, and hot-compressed/humid-aged CABS glasses. Plastic compressibility (*β*_hot_.), i.e., the relative increase in density upon hot compression, is also given. Coloring indicates density or plastic compressibility value of reference glass or within ±0.2% (grey), increasing density or plastic compressibility relative to reference (green), and decreasing density or plastic compressibility relative to reference (red). The error in density was within ±0.002 g cm^−3^.

Glass ID	*ρ_as-made_* (g cm^−3^)	*ρ_hot_* (g cm^−3^)	*ρ_aging_* (g cm^−3^)	*ρ_hot-aging_* (g cm^−3^)	*β_hot_* (GPa^−1^)
CABS-ref	2.411	2.556	2.430	2.563	6.01
CABS-SiB	2.424	2.552	2.403	2.541	5.28
CABS-CaB	2.539	2.634	2.541	2.637	3.74
CABS-BAl	2.395	2.515	2.388	2.515	5.01
CABS-CaSi	2.497	2.640	2.503	2.635	5.73
CABS-CaAl	2.499	2.603	2.506	2.607	4.16

**Table 3:** Young’s moduli (*E*) of the as-made, hot-compressed, humid-aged, and hot-compressed/humid-aged CABS glasses. Coloring indicates Young’s moduli of reference glass (grey), increasing Young’s moduli relative to reference (green), and decreasing Young’s moduli relative to reference (red). The error was within ±2 GPa.

Glass ID	*E_as-made_* (GPa)	*E_hot_* (GPa)	*E_aging_* (GPa)	*E_hot-aging_* (GPa)
CABS-ref	67	81	72	83
CABS-SiB	65	78	70	80
CABS-CaB	73	85	77	82
CABS-BAl	63	76	67	78
CABS-CaSi	72	86	72	87
CABS-CaAl	71	80	76	81

**Table 4:** Vickers hardness (*H*_V_) of the as-made, hot-compressed, humid-aged, and hot-compressed/humid-aged CABS glasses. Coloring indicates *H*_V_ value of reference glass (grey), increasing *H*_V_ relative to reference (green), and decreasing *H*_V_ relative to reference (red). The error in hardness was within ±0.2 GPa.

Glass ID	*H*_V *as-made*_ (GPa)	*H*_V *hot*_ (GPa)	*H*_V *aging*_ (GPa)	*H*_V *hot-aging*_ (GPa)
CABS-ref	5.82	6.96	5.70	6.88
CABS-SiB	5.54	7.31	5.97	6.96
CABS-CaB	5.62	7.39	6.11	7.66
CABS-BAl	5.58	6.72	5.50	6.69
CABS-CaSi	5.81	7.14	5.94	7.41
CABS-CaAl	6.11	7.28	6.25	7.32

**Table 5:** Crack resistance (CR) for Vickers indentation of the as-made, hot-compressed, humid-aged, and hot-compressed/humid-aged CABS glasses. Coloring indicates CR value of reference glass (grey), and decreasing CR relative to reference (red). The error in CR was within 15%.

Glass ID	*CR _as-made_* (N)	*CR _hot_* (N)	*CR _aging_* (N)	*CR _hot-aging_* (N)
CABS-ref	13.0	5.6	5.4	4.4
CABS-SiB	13.6	7.8	6.1	3.9
CABS-CaB	5.6	3.8	2.9	2.8
CABS-BAl	18.9	8.0	5.0	3.4
CABS-CaSi	14.5	8.4	8.1	4.6
CABS-CaAl	8.2	6.9	3.9	2.6

**Table 6:** Crack resistance (CR) for cube corner indentation of the as-made, hot-compressed, humid-aged, and hot-compressed/humid-aged CABS glasses. Coloring indicates CR value of reference glass (grey), and decreasing CR relative to reference (red).

Glass ID	*CR _as-made_* (N)	*CR _hot_* (N)	*CR _aging_* (N)	*CR _hot-aging_* (N)
CABS-ref	0.58	0.46	0.15	0.12
CABS-SiB	0.96	0.54	0.28	0.11
CABS-CaB	0.82	0.33	0.27	0.14
CABS-BAl	0.43	0.38	0.16	0.07
CABS-CaSi	0.52	0.25	0.21	0.13
CABS-CaAl	0.51	0.27	0.20	0.06

**Table 7:** Indentation side recovery (*L*_SR_) for the as-made calcium aluminoborosilicate glasses. The reported *L*_SR_ value and corresponding error are based on results from ten independent indentations.

Glass ID	CABS-Ref	CABS-SiB	CABS-CaB	CABS-BAl	CABS-CaSi	CABS-CaAl
*L*_SR_ (%)	35.9	33.7	29.7	37.3	34.3	30.9
Error	1.8	1.9	1.8	1.2	2.0	1.8

## Data Availability

The data presented in this study are available on reasonable request from the corresponding author.

## Supplementary Materials

The following are available online at https://www.mdpi.com/article/10.3390/ma14133450/s1, Figure S1: X-ray diffraction spectra of the calcium aluminoborosilicate (CABS) glasses. Figure S2: Crack probability as a function of applied indentation load for the calcium aluminoborosilicate (CABS) glasses. Figure S3: Differential scanning calorimetry heating scans of the calcium aluminoborosilicate (CABS) glasses. Table S1: Overview of the properties of the pristine glasses, including glass transition temperature (*T*_g_), density (*ρ*), Young’s modulus (*E*), shear modulus (*G*), bulk modulus (*B*), Poisson’s ratio (*ν*), Vickers hardness (*H*_v_), and crack resistance (CR). Table S2: Atomic packing density (*C_g_*) of the as-made, hot compressed, humid aged, and hot-compressed/humid-aged CABS glasses.
