# Supplementary material for: Indentation Response of Calcium Aluminoborosilicate Glasses Subjected to Humid Aging and Hot Compression
Source: Materials (Basel). 2021 Jun 22;14(13):3450. doi: 10.3390/ma14133450 (PMC8269477; doi:10.3390/ma14133450)

Indentation Response of Calcium Aluminoborosilicate Glasses Subjected to Humid Aging and Hot Compression

Xiangting Ren [1], Pengfei Liu [1], Sylwester J. Rzoska [2], Boleslaw Lucznik [2], Michal Bockowski [2] and Morten M. Smedskjaer [1,*]

[1] Department of Chemistry and Bioscience, Aalborg University, 9220 Aalborg, Denmark; xiangtingr@bio.aau.dk (X.R.); pli@bio.aau.dk (P.L.)
[2] Institute of High-Pressure Physics, Polish Academy of Sciences, 01-142 Warsaw, Poland; sylwester.rzoska@gmail.com (S.J.R.); bolo@unipress.waw.pl (B.L.); bocian@unipress.waw.pl (M.B.)
* Correspondence: mos@bio.aau.dk

Citation: Ren, X.; Liu, P.; Rzoska, S.J.; Lucznik, B.; Bockowski, M.; Smedskjaer, M.M. Indentation Response of Calcium Aluminoborosilicate Glasses Subjected to Humid Aging and Hot Compression. *Materials* **2021**, *14*, x. https://doi.org/10.3390/xxxxx

Academic Editor(s): Halina Kaczmarek

Received: date
Accepted: date
Published: date

Publisher's Note: MDPI stays neutral with regard to jurisdictional claims in published maps and institutional affiliations.

Copyright: © 2021 by the authors. Submitted for possible open access publication under the terms and conditions of the Creative Commons Attribution (CC BY) license (http://creativecommons.org/licenses/by/4.0/).

Supporting information

Table S1. Overview of the properties of the pristine glasses, including glass transition temperature (T_g), density (ρ), Young's modulus (E), shear modulus (G), bulk modulus (B), Poisson's ratio (ν), Vickers hardness (H_v), and crack resistance (CR).

Glass ID	T_g [°C]	ρ [g/cm^3]	E [GPa]	G [GPa]	B [GPa]	ν [-]	H_v (1) [GPa]	CR (2) (Vickers) [N]	CR (2) (cube) [N]
CABS-ref	677	2.411	67	26	47	0.260	5.82	13.0	0.5
CABS-SiB	652	2.424	65	26	47	0.267	5.54	13.6	1.0
CABS-CaB	683	2.539	73	28	55	0.275	5.62	5.6	0.8
CABS-BAl	636	2.395	63	25	42	0.249	5.58	18.9	0.4
CABS-CaSi	668	2.497	72	28	51	0.261	5.81	14.5	0.5
CABS-CaAl	665	2.499	71	28	46	0.244	6.11	8.2	0.5

(1) H_V measured at ambient conditions at load of 9.8 N (1 kgf).

(2) CR measured at ambient conditions (temperature 23.5±1 °C, relative humidity ~37%).

Table S2. Atomic packing density (C_g) of the as-made, hot compressed, humid aged, and hot-compressed/humid-aged CABS glasses. Coloring indicates reference density (grey), increasing density relative to reference (green), and decreasing density relative to reference (red). The error in C_g is within ±0.001.

Glass ID	$C_{g\ as\text{-}made}$	$C_{g\ hot}$	$C_{g\ aging}$	$C_{g\ hot\text{-}aging}$
CABS-ref	0.434	0.461	0.438	0.462
CABS-SiB	0.436	0.459	0.433	0.457
CABS-CaB	0.453	0.470	0.454	0.470
CABS-BAl	0.424	0.445	0.423	0.446
CABS-CaSi	0.444	0.470	0.446	0.469
CABS-CaAl	0.438	0.456	0.439	0.457

Figure S1. X-ray diffraction spectra of the calcium aluminoborosilicate (CABS) glasses.

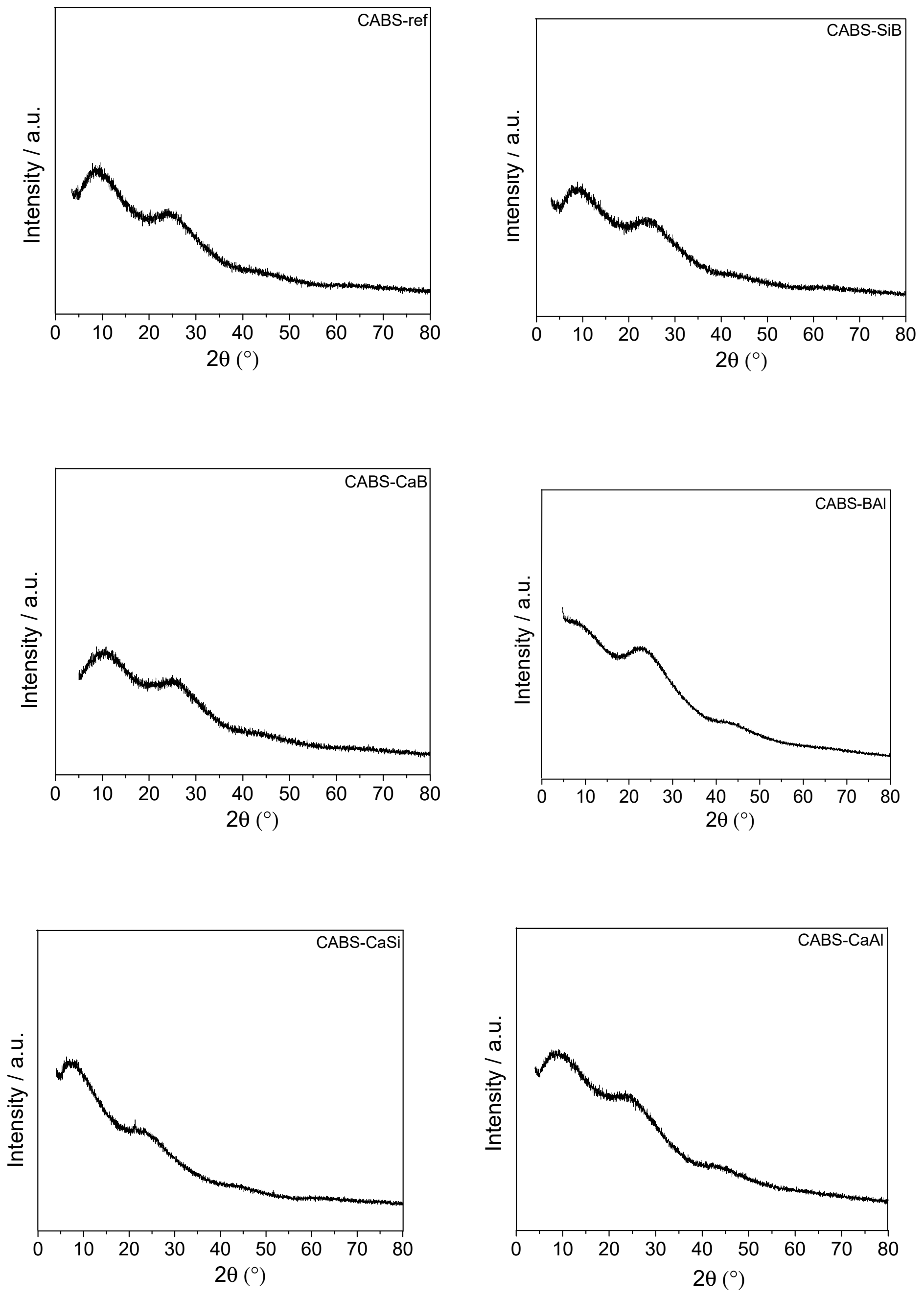

Figure S2. Crack probability as a function of applied indentation load for the calcium aluminoborosilicate (CABS) glasses. The experimental data was fit to a sigmoidal function of the form $y = A_2 + (A_1 - A_2)/[1 + (x/x_0)^p]$ (solid lines).

a. CABS (Hot compression) – Vickers indenter

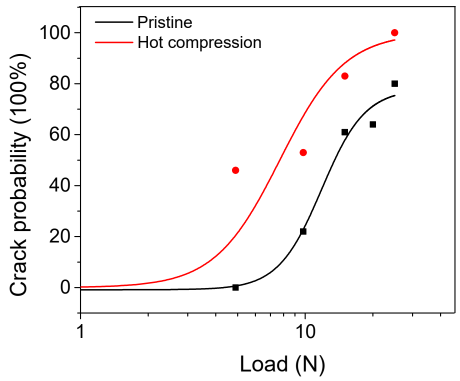

CABS-ref

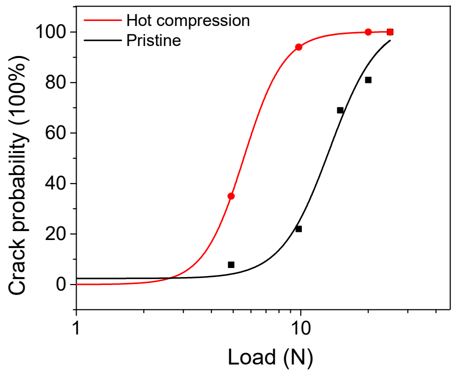

CABS-SiB

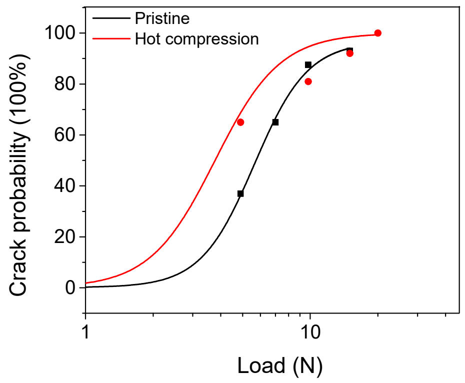

CABS-CaB

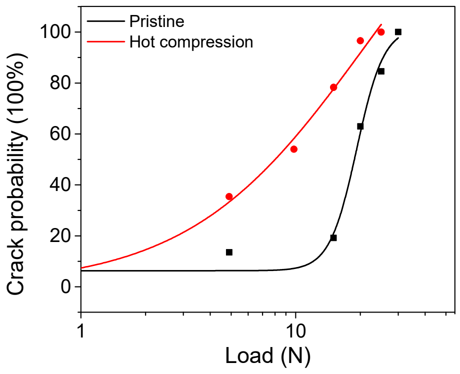

CABS-BAl

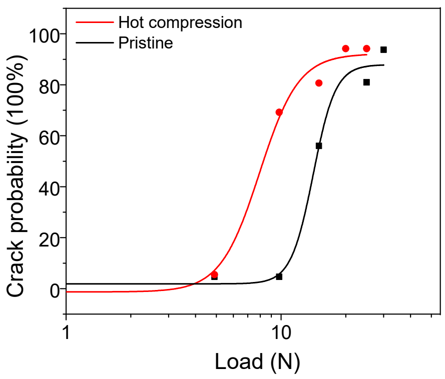

CABS-CaSi

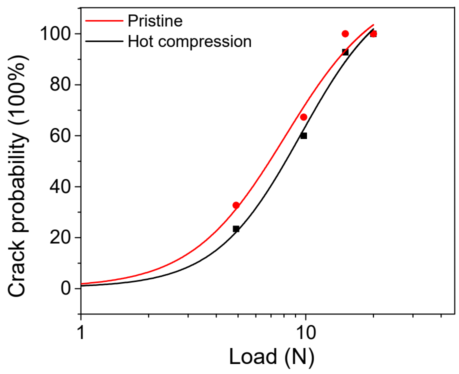

CABS-CaAl

b. CABS (Hot compression) – cube corner indenter

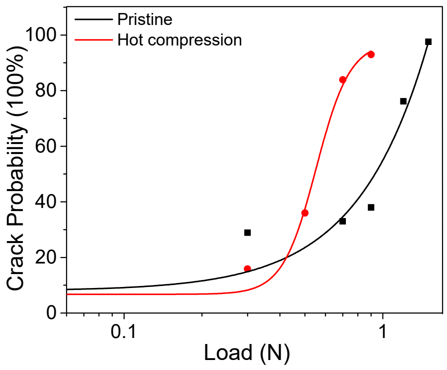

CABS-ref

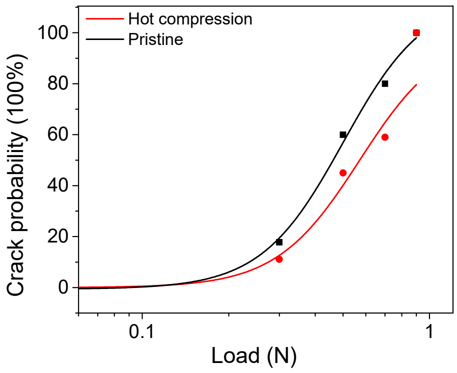

CABS-SiB

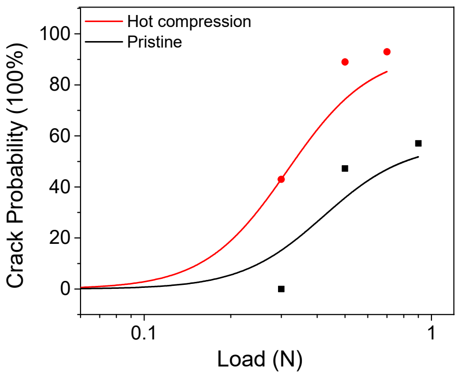

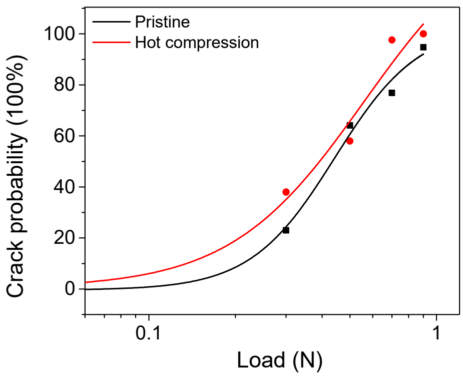

CABS-CaB

CABS-BAl

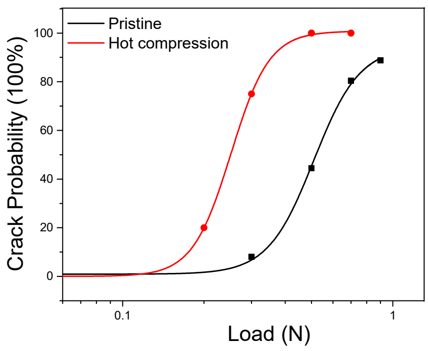

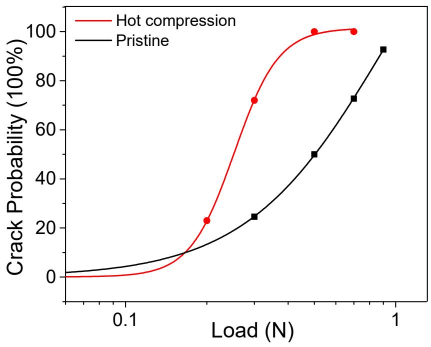

CABS-CaSi

CABS-CaAl

c. CABS (Humid Aging) – Vickers indenter

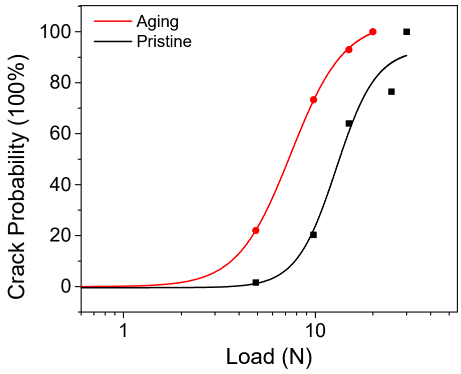

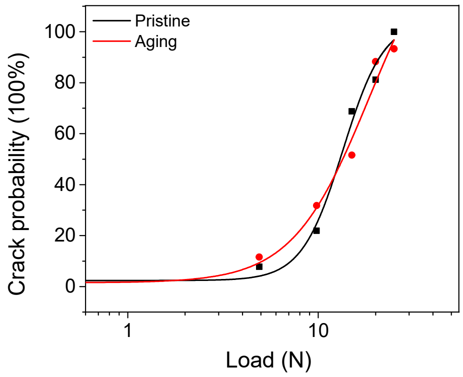

CABS-ref **CABS-SiB**

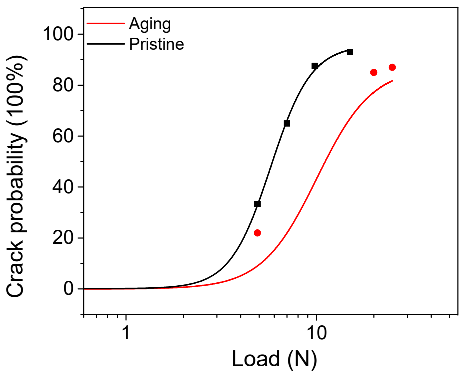

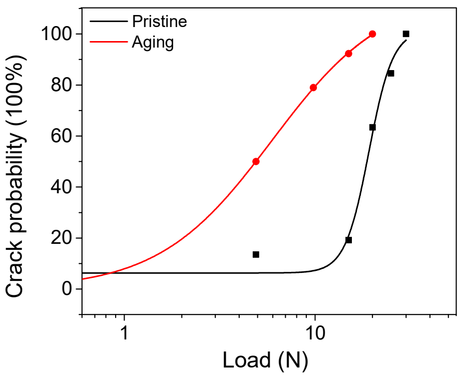

CABS-CaB **CABS-BAl**

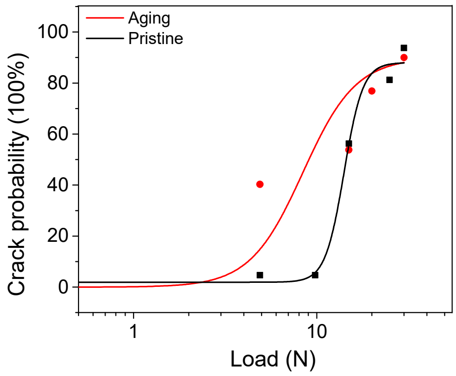

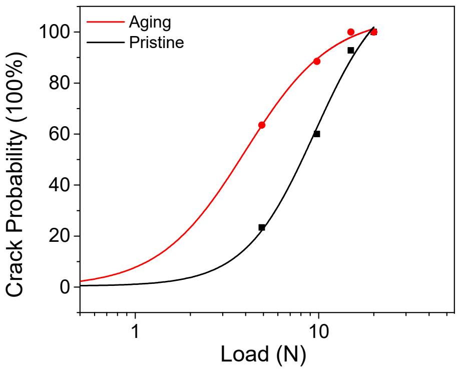

CABS-CaSi **CABS-CaAl**

d. CABS (Humid Aging) - cube corner indenter

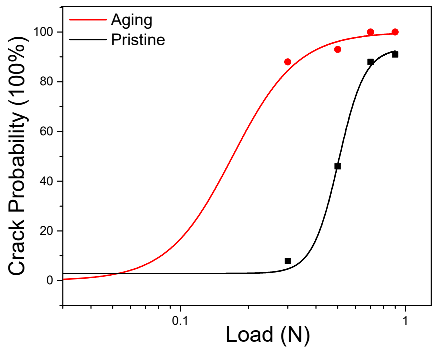

CABS-ref

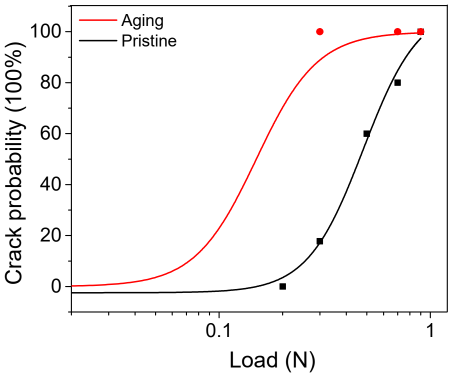

CABS-SiB

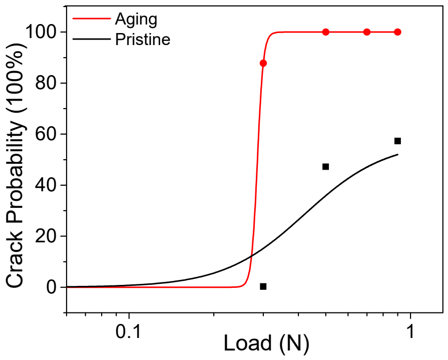

CABS-CaB

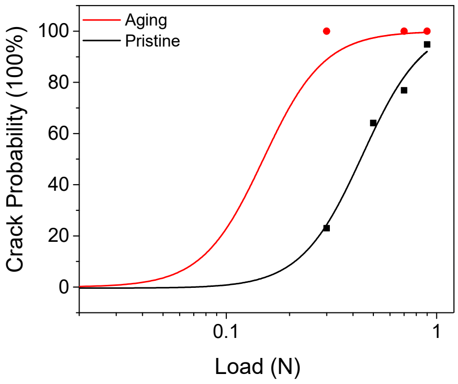

CABS-BAl

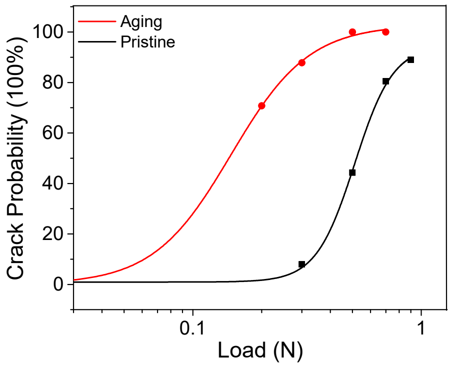

CBAS-CaSi

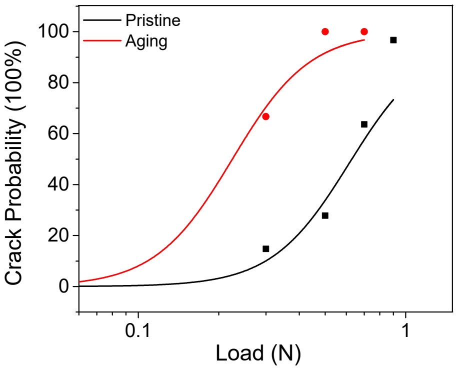

CABS-CaAl

e. CABS (Hot-Aging) – Vickers indenter

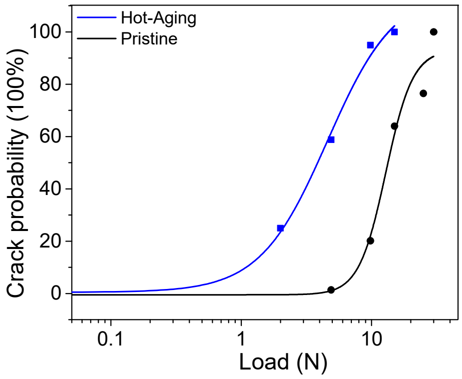

CABS-ref

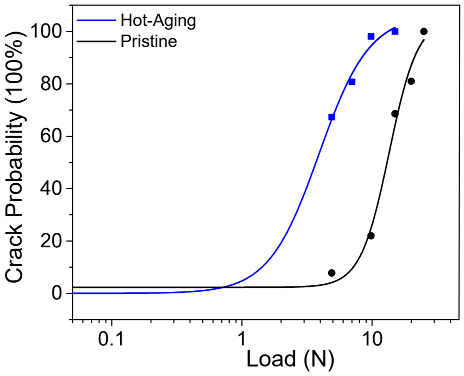

CABS-SiB

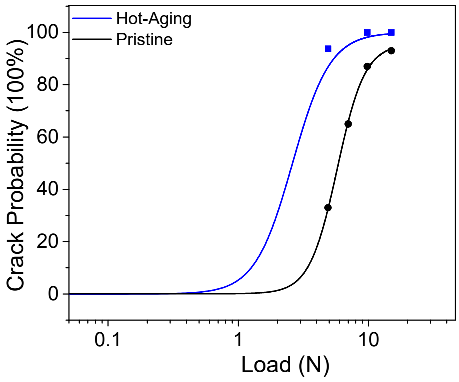

CABS-CaB

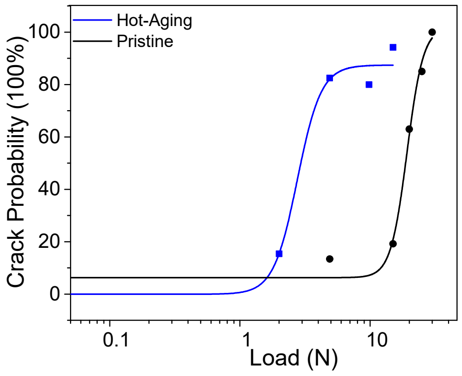

CABS-BAl

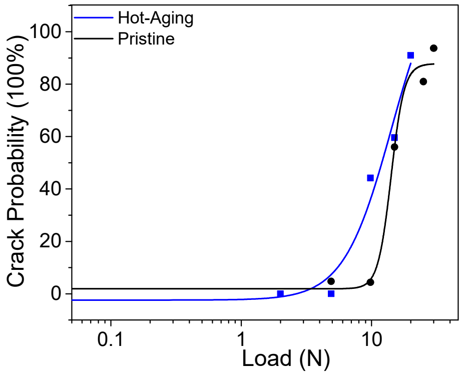

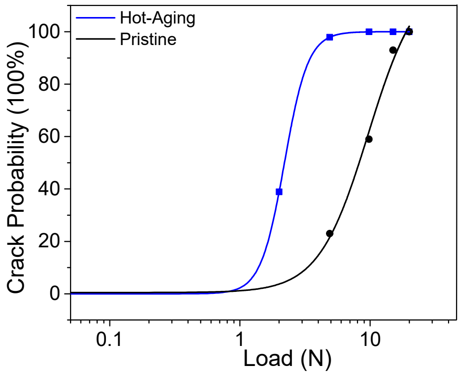

CABS-CaSi **CABS-CaAl**

f. CABS (Hot-Aging) – cube corner indenter

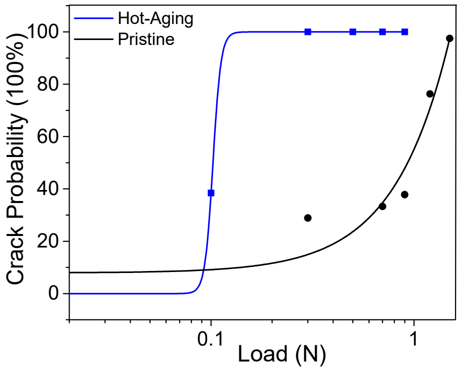

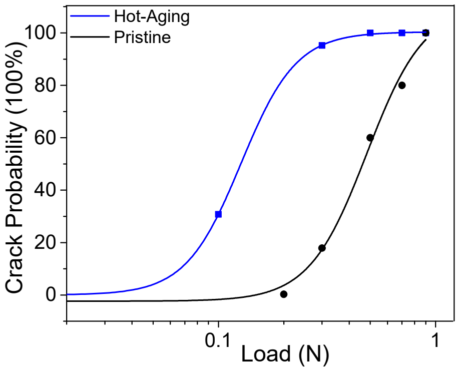

CABS-ref **CABS-SiB**

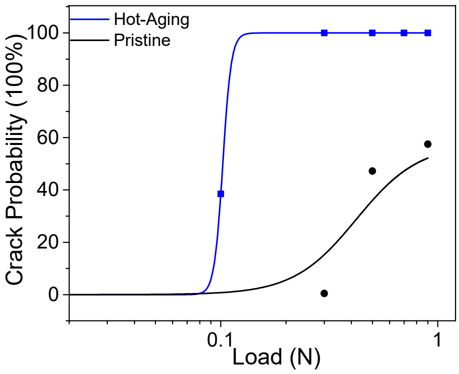

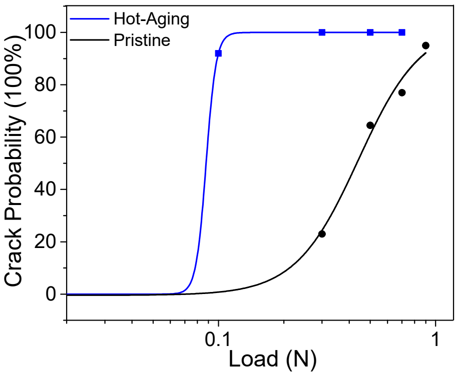

CABS-CaB **CABS-BAl**

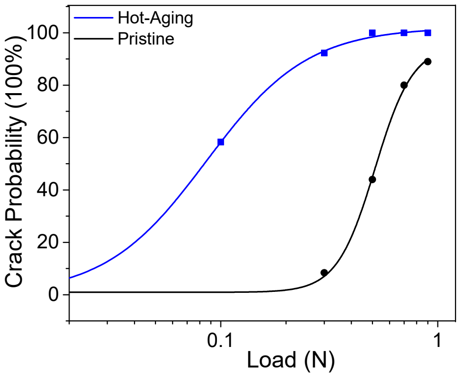

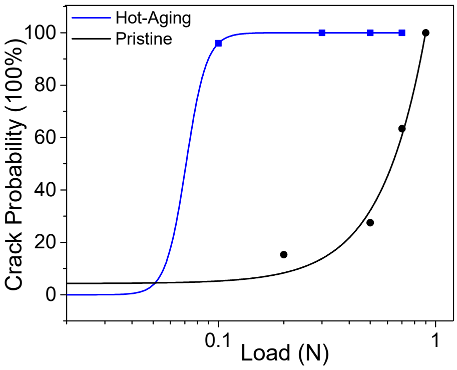

CABS-CaSi **CABS-CaAl**

Figure S3. Differential scanning calorimetry heating scans of the calcium aluminoborosilicate (CABS) glasses.

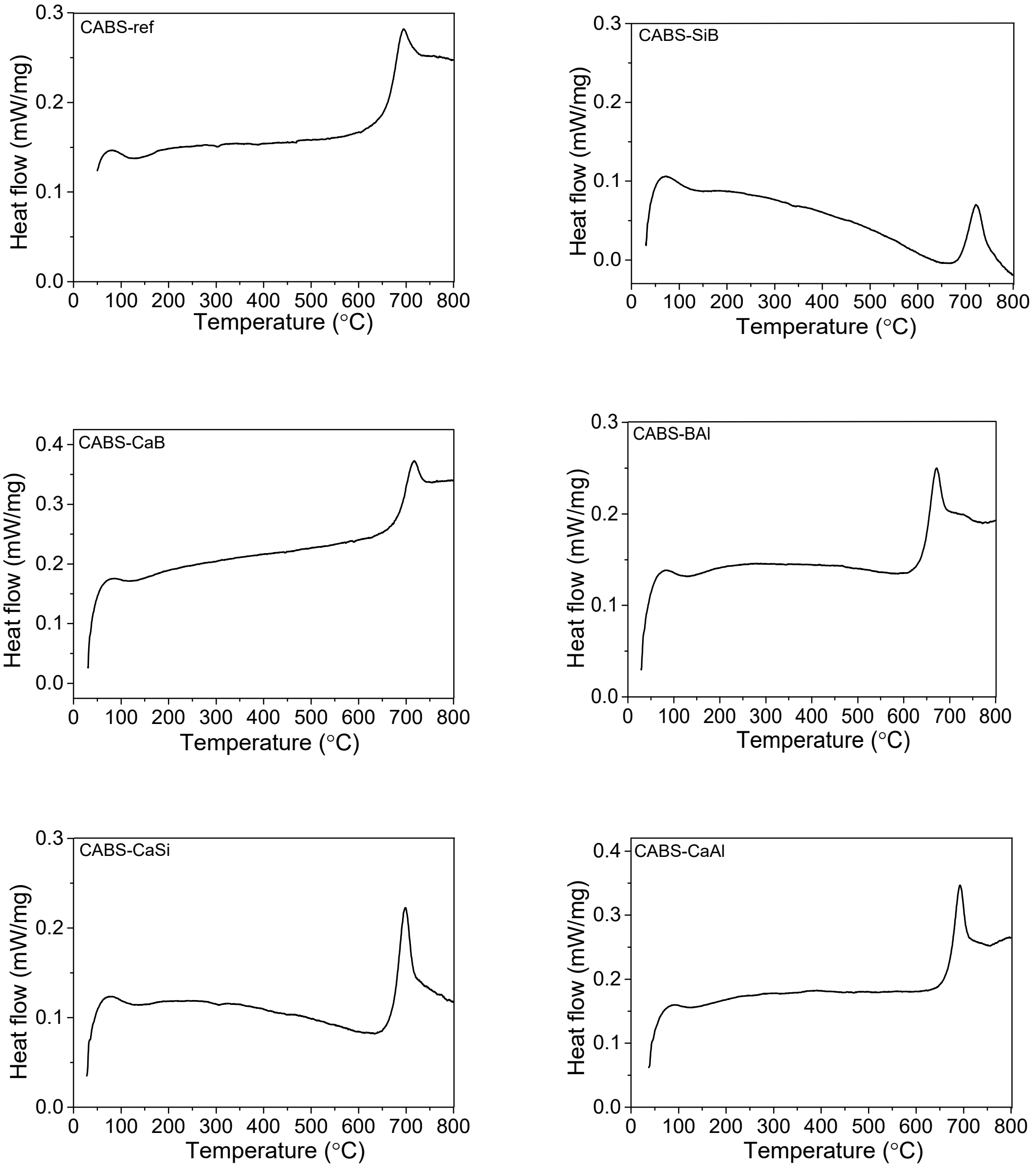

Supplement: Supplementary file 1 [file materials-14-03450-s001.zip › materials-1236278-supplementary.pdf]
